# Supplementary material for: Longitudinal observational study of boxing therapy in Parkinson’s disease, including adverse impacts of the COVID-19 lockdown
Source: BMC Neurol. 2021 Aug 24;21:326. doi: 10.1186/s12883-021-02359-6 (PMC8382938; doi:10.1186/s12883-021-02359-6)
Supplement: Supplementary file 1 — Additional file 1. Supplementary Table 1: Analysis of GEE Parameter Estimates: Empirical Standard Error Estimates. Modeling was based on the absolute number of falls per month, excluding those who reported no falls at any time during the study period. Supplementary Table 2: Analysis of GEE Parameter Estimates: Contrast Estimate Results. Modeling was based on the absolute number of falls per month, excluding those who reported no falls at any time during the study period. Supplementary Table 3: Analysis of GEE Parameter Estimates: Empirical Standard Error Estimates. Modeling was based on the number of months in which at least one fall occurred, excluding those who reported no falls at any time during the study period. Supplementary Table 4: Analysis of GEE Parameter Estimates: Contrast Estimate Results. Modeling was based on the number of months in which at least one fall occurred, excluding those who reported no falls at any time during the study period. [file 12883_2021_2359_MOESM1_ESM.docx]

**Supplementary Data for**

**Longitudinal observational study of boxing therapy in Parkinson’s Disease, including adverse impacts of the COVID-19 lockdown**

Craig Horbinski,^1,2^ Katelyn B. Zumpf,^3^ Kathleen McCortney,^2^ and Dean Eoannou^4^

^1^Department of Pathology, Feinberg School of Medicine, Northwestern University, Chicago, IL 60611

^2^Department of Neurosurgery, Feinberg School of Medicine, Northwestern University, Chicago, IL 60611

^3^Department of Preventative Medicine, Feinberg School of Medicine, Northwestern University, Chicago, IL 60611

^4^Parkinson’s Boxing, Kenmore, NY 14217

| **parameter** |  | **estimate** | **standard error** | **95% confidence limits** | | **Z** | **Pr > \|Z\|** |
| --- | --- | --- | --- | --- | --- | --- | --- |
| **intercept** |  | -1.5905 | 0.4895 | -2.5499 | -0.6311 | -3.25 | 0.0012 |
| **self-reported average number of falls pre-BT** |  | 0.0378 | 0.0208 | -0.0030 | 0.0786 | 1.82 | 0.0693 |
| **months from baseline** |  | 0.0161 | 0.0119 | -0.0072 | 0.0394 | 1.35 | 0.1761 |
| **indicator for lockdown period** | 0 | 0.2072 | 0.3040 | -0.3886 | 0.8031 | 0.68 | 0.4955 |
| **indicator for lockdown period** | 1 | 0.0000 | 0.0000 | 0.0000 | 0.0000 | . | . |
| **months from start of lockdown follow-up** |  | 0.4180 | 0.0895 | 0.2426 | 0.5934 | 4.67 | <.0001 |
| **indicator for return to BT after lockdown** | 0 | -1.0523 | 0.3053 | -1.6507 | -0.4540 | -3.45 | 0.0006 |
| **indicator for return to BT after lockdown** | 1 | 0.0000 | 0.0000 | 0.0000 | 0.0000 | . | . |
| **months since return to BT after lockdown** |  | -0.2023 | 0.1048 | -0.4077 | 0.0032 | -1.93 | 0.0537 |

**Supplementary Table 1: Analysis of GEE Parameter Estimates: Empirical Standard Error Estimates.** Modeling was based on the absolute number of falls per month, excluding those who reported no falls at any time during the study period.

| **label** | **mean estimate** | **mean** | | **chi-square** | **Pr > ChiSq** |
| --- | --- | --- | --- | --- | --- |
|  |  | **confidence limits** | |  |  |
| **slope for BT** | 1.0162 | 0.9928 | 1.0402 | 1.83 | 0.1761 |
| **slope for lockdown** | 1.5436 | 1.2966 | 1.8375 | 23.82 | <.0001 |
| **slope for return** | 0.8301 | 0.6785 | 1.0157 | 3.27 | 0.0705 |
| **slope for return vs lockdown** | 0.5378 | 0.4088 | 0.7076 | 19.63 | <.0001 |
| **slope for lockdown vs BT** | 1.5189 | 1.2746 | 1.8102 | 21.82 | <.0001 |
| **slope for return vs BT** | 0.8169 | 0.6652 | 1.0032 | 3.72 | 0.0537 |

**Supplementary Table 2: Analysis of GEE Parameter Estimates: Contrast Estimate Results.** Modeling was based on the absolute number of falls per month, excluding those who reported no falls at any time during the study period.

| **parameter** |  | **estimate** | **standard error** | **95% confidence limits** | | **Z** | **Pr > \|Z\|** |
| --- | --- | --- | --- | --- | --- | --- | --- |
| **intercept** |  | -1.8763 | 0.4672 | -2.7920 | -0.9606 | -4.02 | <.0001 |
| **self-reported average number of falls pre-BT** |  | 0.0342 | 0.0173 | 0.0003 | 0.0681 | 1.98 | 0.0480 |
| **months from baseline** |  | 0.0092 | 0.0098 | -0.0099 | 0.0283 | 0.94 | 0.3474 |
| **indicator for lockdown period** | 0 | 0.4002 | 0.3390 | -0.2642 | 1.0647 | 1.18 | 0.2377 |
| **indicator for lockdown period** | 1 | 0.0000 | 0.0000 | 0.0000 | 0.0000 | . | . |
| **months from start of lockdown follow-up** |  | 0.3737 | 0.0926 | 0.1922 | 0.5553 | 4.03 | <.0001 |
| **indicator for return to BT after lockdown** | 0 | -1.1973 | 0.2931 | -1.7718 | -0.6227 | -4.08 | <.0001 |
| **indicator for return to BT after lockdown** | 1 | 0.0000 | 0.0000 | 0.0000 | 0.0000 | . | . |
| **months since return to BT after lockdown** |  | -0.2275 | 0.0874 | -0.3988 | -0.0562 | -2.60 | 0.0092 |

**Supplementary Table 3: Analysis of GEE Parameter Estimates: Empirical Standard Error Estimates.** Modeling was based on the number of months in which at least one fall occurred, excluding those who reported no falls at any time during the study period.

| **label** | **mean estimate** | **mean** | | **chi-square** | **Pr > ChiSq** |
| --- | --- | --- | --- | --- | --- |
|  |  | **confidence limits** | |  |  |
| **slope for BT** | 1.0092 | 0.9901 | 1.0287 | 0.88 | 0.3474 |
| **slope for lockdown** | 1.4665 | 1.2257 | 1.7547 | 17.50 | <.0001 |
| **slope for return** | 0.8038 | 0.6791 | 0.9516 | 6.44 | 0.0112 |
| **slope for return vs lockdown** | 0.5481 | 0.4260 | 0.7052 | 21.87 | <.0001 |
| **slope for lockdown vs BT** | 1.4531 | 1.2119 | 1.7424 | 16.28 | <.0001 |
| **slope for return vs BT** | 0.7965 | 0.6711 | 0.9453 | 6.78 | 0.0092 |

**Supplementary Table 4: Analysis of GEE Parameter Estimates: Contrast Estimate Results.** Modeling was based on the number of months in which at least one fall occurred, excluding those who reported no falls at any time during the study period.
